# Mechanistic insight into the frequency-dependent ultrasound-assisted extraction of *Rosa laevigata* Polysaccharides: Structure, antioxidant activity, and process optimization

**DOI:** 10.1016/j.ultsonch.2025.107577

**Published:** 2025-09-19

**Authors:** Yuyuan Duan, Shuting Wang, Xiaorong Zhang, Huimei Zhang, Huizhu Wang, Shuai Chen

**Affiliations:** a College of Chemistry and Pharmaceutical Engineering, Jilin University of Chemical Technology, Jilin 132022, China; b Graduate School, Jilin University of Chemical Technology, Jilin 132022, China

**Keywords:** Ultrasound-assisted extraction, Ultrasonic frequency, *Rosa laevigata* polysaccharide, Antioxidant activity, Support vector regression

## Abstract

*Rosa laevigata* Michx. polysaccharide (RLMP) possess considerable bioactive potential; however, the frequency-dependent mechanisms underlying their ultrasound-assisted extraction (UAE) remain insufficiently understood. This study systematically investigated the influence of ultrasonic frequency on the extraction efficiency, structural characteristics, and antioxidant activity of RLMP, with the goal of optimizing the UAE process. Single-frequency ultrasound (SFU) at 20, 40, and 53 kHz and dual-frequency ultrasound (DFU) at 20/40 and 20/53 kHz were compared. Results demonstrated that 40 kHz SFU achieved the highest extraction yield, accompanied by higher glucose content, favorable molecular weight, and reduced protein contamination. In contrast, DFU treatments resulted in lower yields and elevated protein levels, indicating destructive wave interference rather than synergistic effects. Structural analyses further revealed that ultrasonic frequency significantly influenced monosaccharide composition, molecular weight distribution, and surface morphology, while the primary chemical structure remained unchanged. RLMP extracted at 40 kHz exhibited superior antioxidant activity, with more effective scavenging of diphenyl-1-picrylhydrazyl, 2,2′-azino-bis(3-ethylbenzothiazoline-6-sulfonic acid), and superoxide anion radicals. Cellular antioxidant assays further confirmed its protective effects, showing a 42.83 % reduction in intracellular reactive oxygen species and enhanced superoxide dismutase activity. Comparative analyses demonstrated that UAE outperformed both hot-water extraction and microwave-assisted extraction under identical conditions. Process optimization using particle swarm optimization-support vector regression achieved higher predictive accuracy (*R*^2^ = 0.9983) than response surface methodology (*R*^2^ = 0.9816), identifying optimal extraction parameters: 34 min sonication time, 19 mL/g liquid–solid ratio, 180 W power, 41°C temperature, and 0.355 mm particle size, under which a maximum yield of 11.07 % was obtained. The purified RLMP-2 fraction, which contained 93.34 % total carbohydrates content, was characterized by nuclear magnetic resonance (NMR) as a heteropolysaccharide containing both ⍺- and β-glycosidic linkages. These findings provide theoretical guidance for the industrial-scale ultrasonic extraction of RLMP, though challenges including acoustic field uniformity, energy efficiency, and equipment scaling require systematic investigation before commercial implementation.

## 1 Introduction

Rosa laevigata Michx. (RLM), commonly known as the Cherokee rose, is the dried mature fruit of a perennial climbing shrub in the Rosaceae family, widely cultivated in southeastern and southwestern China. Traditionally, RLM has been employed in Chinese medicine to treat gynecological and urological disorders, including urinary frequency, menstrual irregularities, uterine prolapse, and leucorrhea [1]. These applications are well documented in classical Chinese pharmacopeias and continue to hold significance in contemporary herbal practice.

In recent years, research has increasingly focused on elucidating the pharmacological activities of RLM. Contemporary studies have demonstrated its broad spectrum of bioactivity, including immunomodulatory, antioxidant, antidiabetic, antihyperlipidemic, and hepatoprotective properties [[2], [3], [4], [5]]. Polysaccharides, which constitute approximately 25 % of the fruit's dry weight, are recognized as the principal bioactive macromolecules responsible for these effects. Notably, these compounds exhibit diverse health-promoting activities, particularly anti-inflammatory, anti-obesity, and prebiotic functions, with antioxidant capacity serving as a critical indicator of the biological potential of natural polysaccharides [6].

Various extraction techniques—such as hot water extraction (HWE), enzyme-assisted extraction (EAE), microwave-assisted extraction (MAE), and ultrasound-assisted extraction (UAE)—have been employed to isolate plant-derived polysaccharides [[7], [8], [9]]. Among these methods, UAE has attracted considerable attention owing to its high efficiency and its capacity to preserve thermolabile constituents. These benefits arise from ultrasonic cavitation, which disrupts cell walls and enhances mass transfer, thereby facilitating the release of intracellular components [10,11]. The UAE method is particularly advantageous for extracting *R. laevigata* polysaccharide (RLMP) attributed to the fruit's dense pericarp structure and high pectin content [12], which are amenable to cavitation-induced cell wall disruption. However, most existing extraction studies for RLMP have relied on traditional methods [13,14], leaving the potential of frequency-optimized UAE largely unexplored for this species. The performance of UAE is influenced by acoustic parameters, particularly ultrasonic frequency, which governs cavitation intensity and bubble dynamics, thereby modulating both mechanical disruption and sonochemical effects. Although numerous studies have investigated UAE for polysaccharide extraction, most have focused on optimizing power, time, and temperature parameters [10,11,15]. The effect of ultrasonic frequency on polysaccharide yield and bioactivity, however, remains poorly understood, with few systematic investigations comparing single- and dual-frequency effects. Studies on other plant materials have suggested that extraction selectivity can be frequency-dependent [[16], [17], [18]], yet no comprehensive frequency screening has been reported for RLMP. Moreover, dual-frequency ultrasonic extraction remains largely unexplored for polysaccharide extraction, despite theoretical advantages in cavitation control and extraction selectivity [18,19]. This knowledge gap is particularly important, as ultrasonic frequency fundamentally influences cavitation characteristics, thereby affecting both extraction efficiency and the structural and antioxidant properties of the extracted polysaccharides.

To optimize the extraction of active components from traditional Chinese medicine (TCM), statistical and experimental design tools—such as response surface methodology (RSM), orthogonal experimental design (OED), and uniform design (UD)—are commonly employed to enhance extraction yields. Among these, RSM is favored for its simplicity and its ability to reveal interactions between variables; however, its reliance on quadratic models limits its precision in highly nonlinear systems [20,21]. In contrast, particle swarm optimization-support vector regression (PSO-SVR) offers greater modeling flexibility and predictive accuracy, particularly in complex multivariable contexts [22]. PSO-SVR has been widely applied across various fields, including process optimization, forecasting, parameter optimization, and agriculture and food science, to address complex nonlinear regression and optimization problems [[23], [24], [25]].

The objective of this study was to investigate the frequency-dependent effects of ultrasound on the extraction of RLMP and to elucidate the relationship between ultrasonic frequency and the structural and antioxidant properties of the extracted polysaccharides. Specifically, we aimed to: (1) compare the extraction efficiency of single-frequency (SFU; 20, 40, and 53 kHz) and dual-frequency ultrasound (DFU; 20/40 and 20/53 kHz); (2) characterize the physicochemical and antioxidant properties of frequency-specific extracts; and (3) optimize extraction parameters using RSM and PSO-SVR modeling. This systematic investigation addresses a critical knowledge gap in the mechanisms underlying frequency-specific UAE and provides foundational data for understanding the potential applications of RLMP as natural antioxidant agents.

## 2 Materials and methods

### 2.1 Materials and reagents

Fruits of RLM were procured from local pharmacies in Changchun, Jilin Province, China. Monosaccharide standards—D-mannose (Man), D-glucuronic acid (GlcA), L-rhamnose (Rha), D-galacturonic acid (GalA), D-glucose (Glc), D-galactose (Gal), L-arabinose (Ara), and D-xylose (Xyl)—were purchased from the National Institutes for Food and Drug Control (Beijing, China). Trifluoroacetic acid (TFA), superoxide dismutase (SOD) and malondialdehyde (MDA) assay kits were purchased from Shanghai Macklin Biochemical Co., Ltd. (Shanghai, China). N-acetylcysteine (NAC) was purchased from Sigma-Aldrich Co. LLC (St. Louis, MO, USA). 2,2-Diphenyl-1-picrylhydrazyl (DPPH) and 2,2′-azino-bis(3-ethylbenzothiazoline-6-sulfonic acid) (ABTS) were supplied by Aladdin Chemical Co., Ltd. (Shanghai, China). Diethylaminoethyl cellulose (DEAE-52) was obtain from Whatman International Ltd. (Maidstone, UK). All other chemicals were of analytical grade and used without further purification. Double-distilled water was employed in all experiments.

### 2.2 Multi-frequency ultrasound extraction system

A UAE system was assembled using three ultrasonic instruments: a SCIENTZ-IID ultrasonic cell disruptor (Ningbo Scientz Biotechnology Co., Ltd., China) equipped with a probe-type ultrasonic processor (20–25 kHz, 200 W, 707.4 W/cm^2^); an SK5210HP ultrasonic cleaner (Shanghai Kudos Ultrasonic Instrument Co., Ltd., China) operating at 53 kHz with an output power of 200 W (6.4 W/cm^2^); and a KQ5200DE digital ultrasonic cleaner (Kunshan Ultrasonic Instruments Co., Ltd., China) operating at 40 kHz with power of 80–200 W (8.7 W/cm^2^). The system was configured to operate in either single-frequency mode (20, 40, or 53 kHz) or dual-frequency mode (20/40 or 20/53 kHz).

### 2.3 Extraction of RLMP

Dried *R. laevigata* fruits (200 g) were ground and sieved through a 40-mesh screen. Lipids were removed using petroleum ether. The resulting defatted powder was mixed with deionized water at a 1:20 ratio (g/mL) and subjected to ultrasound treatment at different frequencies: SFU (20, 40, and 53 kHz), DFU (20/40 and 20/53 kHz). Ultrasonication was performed at 200 W for 30 min at 20°C using a water bath circulator. The extract was subsequently cooled, filtered, and concentrated, followed by precipitation with 95 % (v/v) ethanol at a 1:4 ratio (extract:ethanol, v/v), maintained at 4°C for 12 h to obtain crude RLMP. Total carbohydrate content was determined using the phenol–sulfuric acid method with a glucose standard curve (*A* = 42.467*C* + 0.0685, *R*2 = 0.9999). The extraction yield (%) was calculated using the formula:

(1) $$\begin{array}{c} \mathrm{Extractionyield}\left(\%\right)=\frac{C\times V\times d}{m}\times 100\% \end{array}$$

where *C* denotes total carbohydrate content (mg/mL); *V* represents filtrate volume (mL); *d* is the dilution factor; and *m* denotes the weight of raw material (g).

### 2.4 Preliminary characterization

#### 2.4.1 General components

Total carbohydrate content was assessed as described in Section 2.3. Protein content was determined using the Bradford assay, with a bovine serum albumin standard curve (*A* = 0.0444*C* − 0.042, *R*^2^ = 0.9998). Uronic acid (UA) content was quantified using the m-hydroxybiphenyl method [26], based on a galacturonic acid standard curve (*A* = 0.0303*C* − 0.0543, *R*^2^ = 0.9997).

#### 2.4.2 Monosaccharide composition analysis

Monosaccharide composition was analyzed following the method described by Wang, et al. [27], with minor modifications. RLMP (10 mg) was hydrolyzed in 7 mL of 2 M TFA at 110 °C for 5 h. After centrifugation, residual TFA was removed by repeated co-evaporation with methanol. The hydrolysate was derivatized with 1-phenyl-3-methyl-5-pyrazolone (PMP) by mixing 100 μL of the sample with 100 μL of 0.5 M PMP in methanol and 100 μL of 0.3 M NaOH, followed by incubation at 70 °C for 60 min. The reaction mixture was then cooled to room temperature and neutralized with 100 µL of 0.3 M HCl. Subsequently, 5 mL of chloroform was added, and the mixture was vigorously vortexed and centrifuged to remove the organic phase. This extraction was repeated three times. The aqueous phase was diluted to a final volume of 2 mL with distilled water and filtered through a 0.45 µm microporous membrane prior to HPLC analysis. Monosaccharide standards were prepared and processed using the same procedure.

Chromatographic separation was performed using a 1260 HPLC system (Agilent Technologies, Santa Clara, CA, USA) equipped with a ZORBAX Eclipse XDB-C18 column (4.6 × 250 mm, 5 μm). The mobile phase comprised 0.05 M KH_2_PO_4_ and 18 % acetonitrile, delivered at a flow rate of 1.0 mL/min. Detection was conducted at 245 nm. Seven monosaccharide standards were prepared at concentrations ranging from 0.1 to 2.0 mg/mL, and calibration curves were constructed with correlation coefficients (*R*^2^) exceeding 0.999 for all analytes.

#### 2.4.3 Molecular weight (Mw) distribution

The Mw was determined using a Thermo U3000 HPLC system equipped with a BRT 105–103-101 gel column (8 × 300 mm) and a Shimadzu RI-20A refractive index detector. The mobile phase consisted of 0.2 M NaCl, delivered at a flow rate of 0.7 mL/min. The column was maintained at 40 °C, and the injection volume was 50 μL.

#### 2.4.4 Ultraviolet (UV) spectroscopy

A 0.6 mg/mL RLMP solution was analyzed over the 200–400 nm range using a TU-1950 UV–Vis spectrophotometer (Beijing Puxi General Instrument Co., Ltd., China).

#### 2.4.5 Fourier Transform Infrared (FT–IR) spectroscopy

RLMP was mixed with spectroscopic-grade KBr at a 1:100 (w/w) ratio, ground in an agate mortar for 5 min, and pressed into pellets at 10 tons for 30 s. The samples were analyzed using a Nicolet 6700 FT–IR spectrometer (Thermo Fisher Scientific, USA) over the range of 4000–400 cm^−1^.

#### 2.4.6 Scanning electron microscopy (SEM)

The morphology of RLMP was examined using a SU8600 scanning electron microscope (Hitachi High-Technologies Corporation, Tokyo, Japan) operated at an accelerating voltage of 5 kV. Dried RLMP samples (5 mg) were sputter-coated with an ≈ 10 nm Au/Pd alloy film at 20 mA for 60 s under an Ar pressure of 0.08 mbar after reaching a base vacuum of < 5 × 10^-5^ mbar.

#### 2.4.7 Thermogravimetric analysis (TGA)

Thermal stability was evaluated using a TA SDT650 simultaneous thermal analyzer. Although the instrument is capable of simultaneously measuring both mass loss and heat flow, the present study focused solely on thermogravimetric analysis to identify thermal transitions. Approximately 1–2 mg of each sample was heated from 50 °C to 800 °C at a constant rate of 10 °C /min in a nitrogen atmosphere (50 mL/min). The derivative thermogravimetry (DTG) curve, obtained by calculating the first derivative of the TG data, was employed to more clearly identify the characteristic decomposition peaks associated with each stage of mass loss.

### 2.5 Antioxidant activity in cell-free systems

To evaluate the radical-scavenging capacity of the test samples from multiple mechanistic perspectives, three widely accepted cell-free antioxidant assays were conducted: DPPH, ABTS, and superoxide anion radical scavenging assays.

#### 2.5.1 DPPH radical scavenging assay

DPPH scavenging activity was evaluated as described by Jiang, et al. [28]. A 0.1  mL aliquot of the sample solution was mixed with 3.9 mL of DPPH solution, incubated in the dark at room temperature (25 ± 2°C) for 40 min, and the absorbance was measured at 517 nm. Ascorbic acid was used as a positive control. The scavenging rate (*R*) was calculated as:

(2) $$\begin{array}{c} R=\left(1-\frac{A_{s}-A_{b}}{A_{0}}\right)\times 100\% \end{array}$$

where *A_s_* is the sample + DPPH absorbance, *A_b_* denotes the sample blank, and *A_0_* represents the control. All assays were performed in triplicate, and data are expressed as mean ± standard deviation (SD).

#### 2.5.2 ABTS radical scavenging assay

ABTS^+^· was generated by reacting 7 mM ABTS with 2.45 mM potassium persulfate and allowing the mixture to stand in the dark for 12 h [29]. The resulting solution was diluted with PBS (pH 7.4) to an absorbance of 0.70 ± 0.02 at 734 nm. RLMP (1.00 mL) was then mixed with 8.00 mL of the ABTS solution, incubated at room temperature (25 ± 2°C) for 6 min, and the absorbance was measured at 734 nm. Ascorbic acid was used as the positive control. The scavenging rate was calculated according to Eq. (2). Data were collected from three independent experiments and are presented as mean ± SD.

#### 2.5.3 Superoxide anion scavenging assay

Superoxide radicals were generated through pyrogallol autoxidation [30]. RLMP (0–0.4 mg/mL) was combined with 4.5 mL of 50 mM Tris–HCl buffer (pH 8.2, ionic strength 21 mM) and incubated at 37°C for 10 min. The reaction was initiated by adding 0.4 mL of 3 mM pyrogallol and terminated after 4 min with 1.00 mL of 12 mol/L HCl. Absorbance was measured at 325 nm. Ascorbic acid served as the control. Scavenging activity was calculated according to Eq. (2).

### 2.6 Intracellular antioxidant capacity

#### 2.6.1 Cell culture

The murine macrophage cell line RAW 264.7 was provided by the Jilin Provincial Engineering Research Center for Pharmacological and Efficacy Evaluation of Natural Medicines (Changchun, China). RAW 264.7 cells were cultured in Dulbecco′s Modified Eagle Medium (DMEM) supplemented with 10 % fetal bovine serum (FBS), 100 IU/mL penicillin, and 100 µg/mL streptomycin at 37°C in a humidified incubator containing 5 % CO_2_.

#### 2.6.2 Cell viability

Cells were seeded at a density of 5 × 10^4^ cells/well in 96-well plates and allowed to adhere for 6 h before treatment with RLMP (50–800 μg/mL) for 24 h. The cells were subsequently treated with RLMP (50–800 μg/mL) for 24 h, and cell viability was assessed using the CCK-8 assay.

#### 2.6.3 Reactive oxygen species (ROS) measurement

Cells were treated with H_2_O_2_ in the presence or absence of RLMP and subsequently incubated with 10 μM DCFH-DA for 20 min. NAC (200 μg/mL) served as the positive control. Following incubation, the cells were washed three times with phosphate-buffered saline (PBS, pH 7.4) to remove residual extracellular 2′,7′-Dichlorodihydrofluorescein diacetate (DCFH-DA), resuspended in 500 μL of PBS, and analyzed using a NovoCyte flow cytometer (Agilent Technologies, Santa Clara, CA, USA).

#### 2.6.4 SOD activity and MDA content

After 24 h of RLMP (50–400  μg/mL) or NAC (200 μg/mL) treatment, cells were lysed, and the levels of SOD and MDA were quantified using commercial kits, following the manufacturer's instructions.

### 2.7 Optimization of extraction conditions

Single-factor experiments were conducted to investigate the effects of ultrasound time (10–50 min), liquid–solid ratio (10–30 mL/g), ultrasonic power (120–200 W), ultrasonic temperature (20–60 ℃), and particle size (0.25–0.85 mm). Based on the preliminary findings, a Box–Behnken design (BBD) within the framework of RSM was employed to further optimize the extraction parameters. RSM optimization was conducted using Design Expert software (version 13.0.5.0; Stat-Ease Inc., Minneapolis, MN, USA), which applies numerical optimization algorithms, including gradient-based methods, to determine the optimal conditions based on the fitted polynomial model. The coded factor levels (−1, 0, +1) are presented in Table 1.

**Table 1:** Independent variables and levels of the Box-Behnken Design.

Independent Variable	Levels		
	−1	0	+1
Ultrasound time (min)/X_1_	20	30	40
Liquid-solid ratio (mL/g)/X_2_	15	20	25
Ultrasonic power (W)/X_3_	160	180	200
Ultrasonic temperature (℃)/X_4_	30	40	50
Particle size (mm)/X_5_	0.25	0.55	0.85

### 2.8 Support vector regression (SVR) modeling

#### 2.8.1 Theory

**Fig. 1:**
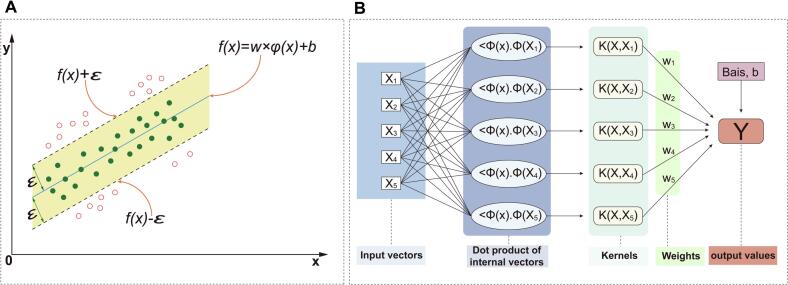
Geometric interpretation of SVR (A); workflow of SVR model construction (B).

SVR maps nonlinear data from a low-dimensional space to a high-dimensional feature space using kernel functions, enabling the construction of a linear regression model in that space to enhance data fitting. The objective is to identify a regression hyperplane, *f(x) = w⋅Φ(x) + b*, such that most data points lie within an ε-insensitive margin (Fig. 1A). Points falling outside this margin are penalized, balancing model complexity and generalization.

#### 2.8.2 Svr-based optimization of RLMP extraction

An SVR model was developed using experimental data obtained from the BBD design. The SVR architecture comprised input vectors (*X_1_–X_5_*), internal dot products, a kernel function, and an output layer (*Y*), as illustrated in Fig. 1B. Both the input variables (*X*) and the output (*Y*) were normalized using the following equations:

(3) $$\begin{array}{c} X_{\mathrm{norm}}=\frac{X-X_{\min}}{X_{\max}-X_{\min}} \end{array}$$

(4) $$\begin{array}{c} Y_{\mathrm{norm}}=\frac{Y-Y_{\min}}{Y_{\max}-Y_{\min}} \end{array}$$

The dataset was randomly divided into training and test sets in an 8:2 ratio, with index shuffling applied to minimize bias [31,32].

The performance of the SVR model depends on the regularization parameter *C* and the kernel parameter *γ*. The parameter *C* regulates the trade-off between error minimization and model simplicity, whereas *γ* determines the bandwidth of the Gaussian kernel:

(5) $$\begin{array}{c} K\left(X_{i},X_{j}\right)=\exp\left({-\gamma \|X_{i}-X_{j}\|}^{2}\right) \end{array}$$

Particle swarm optimization (PSO) was employed to optimize SVR parameters. PSO utilizes an iterative, population-based search strategy defined as follows:

(6) $$\begin{array}{c} v_{i}^{k+1}=wv_{i}^{k}+c_{1}r_{1}\left(p_{\mathrm{best},i}^{k}-x_{i}^{k}\right)+c_{2}r_{2}\left(g_{\mathrm{best}}^{k}-x_{i}^{k}\right) \end{array}$$

(7) $$\begin{array}{c} x_{i}^{k+1}=x_{i}^{k}+v_{i}^{k+1} \end{array}$$

where *w* denotes the inertia weight, *c_1_* and *c_2_* are the learning factors, and *r_1_* and *r_2_* are uniformly distributed random numbers in the range [0, 1]. The PSO parameters were set as follows: inertia weight *w* = 0.8, cognitive learning factor *c_1_* = 2, and social learning factor *c_2_* = 2. A population of 50 particles was used over 50 iterations with *C*, *γ* ∈ [0.1, 100]. Convergence was defined as a change smaller than 10^-4^ over 10 generations.

Model accuracy was assessed using the coefficient of determination (*R*^2^), root mean square error (RMSE), and mean absolute error (MAE):

(8) $$\begin{array}{c} R^{2}=1-\frac{\sum_{i=1}^{n}{\left(Y_{i}-Y_{i}^{\prime }\right)}^{2}}{\sum_{i=1}^{n}{\left(Y_{i}-\overset{¯}{Y}\right)}^{2}} \end{array}$$

(9) $$\begin{array}{c} \mathrm{RMSE}=\sqrt{\frac{1}{n}\sum_{i=1}^{n}{\left(Y_{i}-Y_{i}^{\prime }\right)}^{2}} \end{array}$$

(10) $$\begin{array}{c} \mathrm{MAE}=\frac{1}{n}\sum_{i=1}^{n}\left|Y_{i}-Y_{i}^{\prime }\right| \end{array}$$

where *Y_i_*, $Y_{i}^{\prime }$, and $\overset{¯}{Y}$ represent actual, predicted, and mean values, respectively.

### 2.9 Comparison of extraction methods

HWE, UAE, and MAE of RLMP were conducted under comparable conditions. HWE was performed according to the method described by Zhang et al. [13], with slight modifications: the extraction was carried out at 100°C for 4 h, using a liquid–solid ratio of 20:1 (v/w). MAE was conducted using an XH-300B ultrasonic–microwave cooperative extraction system (Beijing Xianghu Technology Development Co., Ltd., Beijing, China), following the procedure reported by Gao et al. [33], with minor modifications: microwave power was set to 500 W, extraction time was 20  min, and the liquid–solid ratio was maintained at 20:1 (v/w). UAE was performed at 40 kHz and 180 W for 35 min, and the liquid–solid ratio was set at 20:1 (v/w), and the ultrasonic bath temperature was maintained at 40°C. The total carbohydrate content in the extracts was determined using the phenol–sulfuric acid method with a glucose standard curve (*A* = 42.467*C* + 0.0685, *R*^2^ = 0.9999). The extraction yield of RLMP was calculated according to Eq. (1).

### 2.10 Isolation and purification

#### 2.10.1 Deproteinization and decolorization

Crude RLMP obtained under the optimized UAE conditions was dissolved in distilled water at a 1:2 (w/v) ratio, and proteins were removed using a slightly modified Sevag method, as described by Chen et al. [17]. The Sevag reagent (chloroform:n-butanol = 4:1 [v/v]) was mixed with the RLMP solution at a 1:4 (v/v) ratio in a separatory funnel. The mixture was vigorously shaken at 25°C for 30 min, followed by centrifugation at 8000 rpm (7155 × g) for 15 min to collect the supernatant. This process was repeated three times. The combined supernatants were decolorized by adding 1 % (w/v) activated carbon, followed by stirring in a water bath at 60–80°C for 30 min. The mixture was then centrifuged to remove the activated carbon, and the decolorization procedure was repeated twice. The final supernatant was subsequently freeze-dried.

#### 2.10.2 Purification

Purified RLMP (100 mg) was dissolved in 10  mL of distilled water and loaded onto a DEAE-52 cellulose (diethylaminoethyl cellulose, Whatman, Maidstone, UK) column pre-equilibrated with deionized water. Gradient elution was performed sequentially with 200 mL each of deionized water, 0.1 M NaCl, 0.3 M NaCl, and 0.5 M NaCl at a flow rate of 1.5 mL/min. Fractions of 10 mL were collected using an automatic fraction collector, with a total run time of 533 min. All eluates were analyzed using the phenol–sulfuric acid method. Polysaccharide-containing fractions were pooled, dialyzed (MWCO 1.0 kDa), and freeze-dried.

### 2.11 Nuclear magnetic resonance (NMR)

Approximately 50 mg of the isolated RLMP fraction was dissolved in 0.5 mL of deuterium oxide (D_2_O) and lyophilized. The resulting powder was then redissolved in 0.5 mL of D_2_O and lyophilized a second time. This exchange procedure was repeated to ensure complete replacement of labile protons. The final sample was dissolved in 0.5 mL of D_2_O, and ^1^H and ^13^C NMR spectra were recorded using a Bruker Avance III HD 600 MHz spectrometer (Bruker BioSpin, Rheinstetten, Germany) equipped with a 5 mm TCI cryoprobe operating at a proton resonance frequency of 600.13 MHz (14.1 T). All measurements were performed at 25°C, and chemical shifts were referenced to internal acetone.

### 2.12 Statistical analysis

Data were analyzed using GraphPad Prism 8.0. Statistical comparisons were performed using one-way analysis of variance (ANOVA) followed by Tukey's post hoc test. Prior to ANOVA, data normality was assessed with the Shapiro–Wilk test (*p* > 0.05 for all datasets), and homogeneity of variances was confirmed using Levene's test (*p* > 0.05). Results are presented as mean ± SD. All reported *p*-values are two-tailed unless otherwise stated. Statistical significance was set at α = 0.05, with significance levels defined as *p* < 0.05, *p* < 0.01, and *p* < 0.001.

## 3 Results

### 3.1 Effect of ultrasonic mode and frequency on extraction yield

The influence of ultrasonic frequency and mode on RLMP extraction was assessed using single-frequency (20, 40, and 53 kHz) and dual-frequency (20/40 kHz and 20/53 kHz) ultrasound. The results are summarized in Table 2. Among these treatments, 40 kHz achieved the highest extraction yield (8.47 %), suggesting that this frequency provides optimal cavitation effects and energy transfer, which are essential for disrupting plant cell walls and enhancing polysaccharide release. In contrast, extraction yields at 20 kHz (4.53 %) and 53 kHz (4.30 %) were significantly lower. The reduced efficiency at 20  kHz may be attributed to insufficient cavitation intensity, whereas the lower performance at 53 kHz likely resulted from rapid bubble collapse with limited energy transfer, which weakens cell wall disruption. Notably, DFU did not demonstrate synergistic effects. The 20/40 kHz combination produced the lowest yield (2.79 %), potentially due to destructive wave interference or diminished cavitation efficiency [16,34]. Although the 20/53 kHz treatment achieved a moderate yield (5.85 %), it remained inferior to that obtained with 40 kHz alone. These results suggest that, among the frequencies tested, 40 kHz was the most effective for polysaccharide extraction under our experimental conditions. Moreover, simultaneous multi-frequency ultrasound does not necessarily enhance extraction efficiency and may, in some cases, even hinder it.

**Table 2:** Extraction yield, chemical compositions, and monosaccharide constituents of RLMP using UAE at different frequencies.

Samples	20 KHz	40 KHz	53 KHz	20/40 KHz	20/53 KHz
Extraction yield (%)	4.53 ± 0.16^c^	8.47 ± 0.32^a^	4.3 ± 0.23^c^	2.79 ± 0.13^d^	5.85 ± 0.16^b^
Total carbohydrates content (%)	29.49 ± 1.20^c^	41.52 ± 1.30^a^	26.39 ± 1.10^d^	39.81 ± 0.91^a^	44.58 ± 0.90^b^
Uronic acid content (%)	2.47 ± 0.05^b^	3.02 ± 0.11^a^	2.76 ± 0.12^a^	2.30 ± 0.09^b^	2.42 ± 0.05^b^
Protein content (%)	3.45 ± 0.08^d^	3.04 ± 0.05^c^	3.11 ± 0.08^c^	3.92 ± 0.09^b^	4.32 ± 0.06^a^
Mw (×10^4^ Da)	1.51	1.72	1.40	1.40	1.53
Mn (×10^4^ Da)	1.50	1.69	1.39	1.37	1.51
Monosaccharide composition (molar ratios) (mol%)					
Man	3.95 ± 0.08^c^	4.52 ± 0.06^a^	4.50 ± 0.05^a^	4.34 ± 0.05^ab^	4.22 ± 0.07^b^
Rha	0.93 ± 0.06^a^	0.66 ± 0.02^b^	0.68 ± 0.04^b^	0.94 ± 0.07^a^	1.01 ± 0.08^a^
GlcA	0.87 ± 0.08^a^	0.79 ± 0.03^a^	0.83 ± 0.06^a^	0.91 ± 0.08^a^	0.89 ± 0.13^a^
GalA	1.58 ± 0.12^a^	1.27 ± 0.04^b^	1.31 ± 0.04^b^	1.60 ± 0.11^a^	1.61 ± 0.11^a^
Glc	76.98 ± 0.90^bc^	78.91 ± 0.80^a^	78.41 ± 1.20^ab^	75.93 ± 1.10^c^	75.98 ± 1.20^c^
Gal	5.20 ± 0.12^a^	4.06 ± 0.11^b^	4.14 ± 0.15^b^	5.22 ± 0.19^a^	5.31 ± 0.14^a^
Ara	10.51 ± 0.27^bc^	9.80 ± 0.13^d^	10.14 ± 0.28^cd^	11.07 ± 0.37^a^	10.98 ± 0.28^ab^

Note: Data are represented as mean ± SD (n = 3). Means in the same column with different letters are significantly different according to Tukey’s test (*p* < 0.05).

### 3.2 Effect of ultrasonic frequency on physicochemical properties

As shown in Table 2, single-frequency sonication at 40 kHz yielded RLMP with the highest UA content (3.02 %) and the lowest protein content (3.04 %). In contrast, extracts obtained at 20 kHz and 53 kHz exhibited slightly higher protein levels (3.45 % and 3.11 %, respectively) and lower UA contents (2.47 % and 2.76 %). These results are consistent with the findings of Chen et al. [16], who reported that moderate-frequency ultrasound effectively disrupts plant cell walls while minimizing the co-extraction of non-polysaccharidic impurities. By comparison, dual-frequency sonication significantly increased protein co-extraction, with protein levels reaching 3.92 % at 20/40 kHz and 4.32 % at 20/53 kHz, both significantly higher than those observed under single-frequency treatments (*p* < 0.05). The enhanced cavitation and shear forces generated by combined frequencies likely disrupted polysaccharide–protein associations through mechanical breakdown of non-covalent interactions such as hydrogen bonds and electrostatic forces [15], facilitating the release of protein–polysaccharide complexes and thereby reducing polysaccharide purity. Additionally, UA contents decreased slightly under dual-frequency treatments (2.30 % for 20/40 kHz and 2.42 % for 20/53 kHz), potentially due to partial degradation of UA residues under intensified acoustic energy or dilution resulting from increased co-extraction of other constituents. Collectively, these findings suggest that while DFU may improve extraction efficiency, it simultaneously increases impurity levels, ultimately compromising the purity of the polysaccharide fraction.

### 3.3 Structural characteristics

#### 3.3.1 Monosaccharide composition

**Fig. 2:**
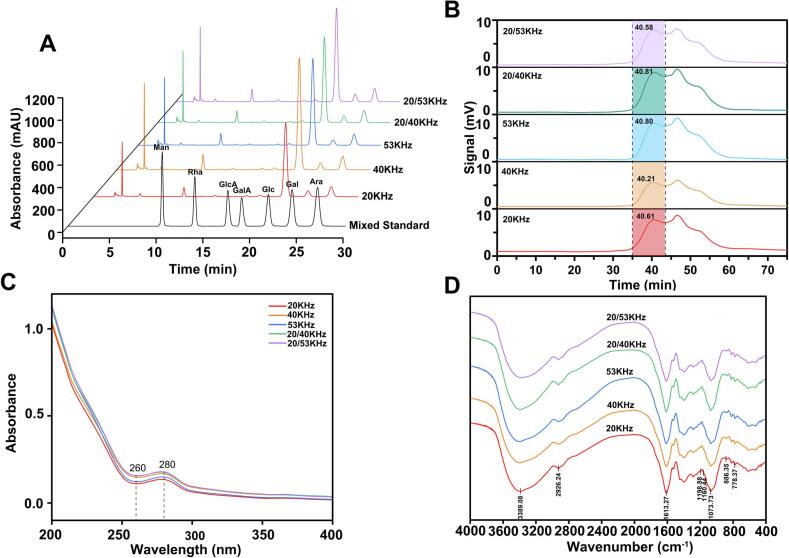
Structural characterization of RLMP obtained under different frequencies. (A) HPLC chromatograms of RLMP samples compared with a mixed monosaccharide standard; (B) HPSEC profiles of RLMP samples; (C) UV–visible spectra (200–400  nm); (D) FT–IR spectra.

The monosaccharide profiles (Fig. 2A) of RLMP extracted under different ultrasonic frequencies exhibited marked variations, highlighting the influence of sonication parameters on cell wall disruption and solubilization. As shown in Table 2, the 40 kHz treatment produced the highest glucose content (78.91 % ± 0.80 %), along with elevated levels of arabinose (9.80 % ± 0.13 %), galactose (4.06 % ± 0.11 %), and mannose (4.52 % ± 0.06 %), indicating more effective cell wall disruption and the release of glucose-rich polysaccharides. The high glucose content is particularly noteworthy, as glucose constitutes the predominant monosaccharide in RLMP [35], making it a reliable indicator for assessing the effectiveness of different extraction conditions. Moreover, glucose serves as the primary backbone unit of many bioactive polysaccharides, which may contribute to enhanced antioxidant activity [36]. In contrast, both the 20 kHz and 53 kHz treatments produced lower monosaccharide yields, likely due to insufficient cavitation at 20 kHz and rapid but less forceful bubble collapse at 53 kHz. Notably, the dual-frequency mode (20/53 kHz) led to increased arabinose (10.98 % ± 0.28 %) and galactose (5.31 % ± 0.14 %) levels, suggesting that combined frequencies may enhance the extraction of arabinogalactan-type polysaccharides with branched structures [35,37]. Nevertheless, the total extraction yield remained lower than that achieved at 40 kHz, indicating that the efficacy of dual-frequency synergy appears to be highly dependent on both the specific frequency combination and the polysaccharide structure [17].

#### 3.3.2 Mw distribution

The Mw distribution of RLMP extracted under different ultrasonic frequency modes was determined using High-Performance Size-Exclusion Chromatography (HPSEC) (Fig. 2B). The results are summarized in Table 2. Under the SFU mode, as the ultrasonic frequency increased from 20 kHz to 40 kHz, Mw rose from 1.51 × 10^4^ Da to 1.72 × 10^4^ Da. However, further increasing the frequency to 53 kHz resulted in a decrease in Mw to 1.40 × 10^4^ Da. This trend can be attributed to the interplay between cavitation intensity and mechanical shear forces. At 40 kHz, the enhanced cavitation effect and moderate shear forces promote efficient extraction while maintaining the integrity of RLMP molecules [11,16], resulting in higher molecular weight. In contrast, at 53 kHz, the reduced molecular weight may be attributed to potentially diminished cavitation intensity due to less effective bubble collapse at higher frequencies [16], combined with possibly excessive shear forces and potential free radical reactions that could contribute to molecular degradation [11,17], leading to a reduction in Mw. In DFU mode, the Mw of RLMP extracted at 20/40 kHz (1.40 × 10^4^ Da) and 20/53 kHz (1.53 × 10^4^ Da) were both lower than that obtained under SFU at 40 kHz, highlighting distinct extraction dynamics between dual-frequency and single-frequency systems. These results indicate that the extraction frequency affects not only the yield but also the molecular characteristics of the extracted polysaccharides.

#### 3.3.3 UV spectroscopy

The UV spectra of RLMP (200–400 nm) are shown in Fig. 2C. A shoulder at 280 nm suggests the presence of residual protein, whereas the absence of absorption at 260 nm indicates minimal nucleic acid content in the samples [18].

#### 3.3.4 FT–IR spectroscopy

The FT–IR spectra of RLMP (Fig. 2D) displayed similar profiles across all samples, suggesting that ultrasound frequency had minimal impact on their fundamental chemical structures. A broad band observed at 3389.08 cm^–^1 was attributed to O–H stretching vibrations, reflecting extensive intra- and intermolecular hydrogen bonding [16]. The moderate-intensity peak at 2926.24 cm^–^1 corresponded to C–H stretching of –CH_3_ and –CH_2_ groups [38]. The absorption at 1613.27 cm^–^1 was assigned to C=O stretching, implying the presence of uronic acid residues [39]. Distinct bands in the range of 1000–1200 cm^–^1 were ascribed to C–O–C and C–OH stretching, characteristic of pyranose ring structures [40]. The peak at 886.35 cm^–^1 indicated β-glycosidic linkages, whereas the band at 778.37 cm^–^1 corresponded to ring deformation vibrations [41,42].

#### 3.3.5 SEM

**Fig. 3:**
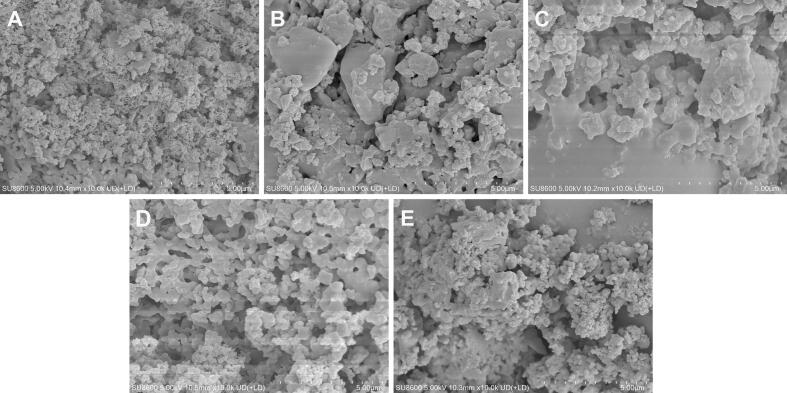
SEM images at magnifications of × 200. (A) 20 kHz, (B) 40 kHz, (C) 53 kHz, (D) 20/40 kHz, and (E) 20/53 kHz.

SEM was employed to examine the surface morphologies of RLMP samples treated under different ultrasonic frequencies (Fig. 3A–E, 10,000 × magnification, scale bar = 5 μm). At 20 kHz, RLMP exhibited compact, cauliflower-like granules with minimal surface disruption (Fig. 3A), indicating that low-frequency cavitation predominantly induced superficial peeling without causing substantial structural damage [16]. When the frequency increased to 40 kHz, the material appeared as smoother, plate-like fragments accompanied by smaller particulate matter (Fig. 3B), suggesting moderate fragmentation resulting from intensified shear forces. At 53 kHz, a loose network of discrete, globular particles was observed (Fig. 3C), likely attributed to enhanced cavitation phenomena—such as microjets and bubble collapse—that promoted the breakdown of larger aggregates into ultrafine fragments, indicative of extensive depolymerization [37]. Under DFU conditions, these effects were further intensified. Specifically, treatment with the 20/40 kHz mode produced a porous, honeycomb-like morphology (Fig. 3D), suggesting synergistic cavitation between the two frequencies that perforated and thinned the polysaccharide sheets [15]. In the 20/53 kHz mode, more severe disruption generated submicron fragments that re-aggregated into loosely packed, fluffy clusters (Fig. 3E), possibly driven by surface energy-mediated recombination. Collectively, these observations demonstrate that increasing the ultrasonic frequency, especially in dual-frequency modes, markedly enhances cavitation intensity and structural disruption. This facilitates glycosidic bond cleavage and contributes to the formation of lower-molecular-weight, structurally modified polysaccharides [18].

#### 3.3.6 TGA

**Fig. 4:**
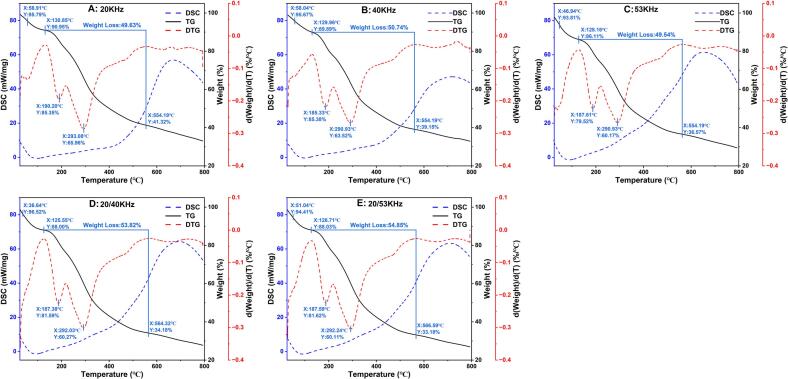
TG, DTG, and DSC curves of RLMP samples obtained at different frequencies: (A) 20 kHz, (B) 40 kHz, (C) 53 kHz, (D) 20/40 kHz, and (E) 20/53 kHz.

Thermogravimetric (TG/DTG) and differential scanning calorimetry (DSC) analyses indicated that all ultrasound-assisted polysaccharide extracts exhibited the characteristic three-step thermal-decomposition profile of plant polysaccharides. As shown in Fig. 4, an initial dehydration phase occurred between 36.64°C and 58.04°C, followed by a minor endothermic transition in the range of 125.55 °C–130.85 °C (*ΔH_T_* 1.82–7.63 kJ/g), which is attributed to the release of loosely bound water [11]. A distinct DTG shoulder at 185.33 °C–190.2 °C (*ΔH_T_* −8.84–-14.31 kJ/g) indicated partial depolymerization and cleavage of side groups, whereas the primary DTG peak, centered at 290.93 °C–293.08 °C (*ΔH_T_* −8.12–-16.33 kJ/g), corresponded to the degradation of the glycosidic backbone. Compared with hot-water-extracted *Curcuma longa* polysaccharides (*T_max_* ≈ 313°C–324°C), the ∼ 20°C reduction in *T_max_* observed for the ultrasound-assisted extracts suggests that acoustic cavitation promotes chain shortening, thereby lowering the activation energy required for thermal decomposition [43]. Furthermore, enhanced carbonization was observed at lower ultrasonic frequencies, with a char yield of 41.32 % at 20 kHz. The higher char yield observed for the 20 kHz extract (41.32 %) can be attributed to the preservation of high-Mw domains and more extensive intermolecular cross-linking, which promote char formation through cyclization and condensation reactions during thermal decomposition [42]. In contrast, the lower char yields obtained under DFU (33.18–34.18 %) are likely the result of intensified glycosidic bond cleavage and chain scission [44], leading to the formation of shorter polysaccharide fragments that are more prone to depolymerization and the generation of volatile products rather than char-forming intermediates.

These differences in thermal stability have important implications for potential applications. Extracts obtained under low-frequency conditions, exhibiting greater thermal resistance, may be more suitable for high-temperature processes such as food extrusion or pharmaceutical tablet compression [45,46]. Conversely, dual-frequency-treated extracts, characterized by lower thermal stability and higher solubility, may be better suited for applications requiring rapid dissolution, such as instant beverage formulations or oral drug delivery systems [47].

### 3.4 Radical scavenging activity of RLMP in cell-free models

Antioxidants play a crucial role in mitigating oxidative stress, a major contributing factor to the development and progression of numerous human diseases [48]. Due to the structural complexity and diverse redox behaviors of plant-derived polysaccharides, their antioxidant properties cannot be comprehensively assessed by a single method. To address this, the radical-scavenging capacities of RLMP were systematically investigated using three complementary cell-free assays—DPPH, ABTS, and superoxide anion scavenging—each targeting distinct mechanisms of antioxidant action.

#### 3.4.1 DPPH radical scavenging activity

**Fig. 5:**
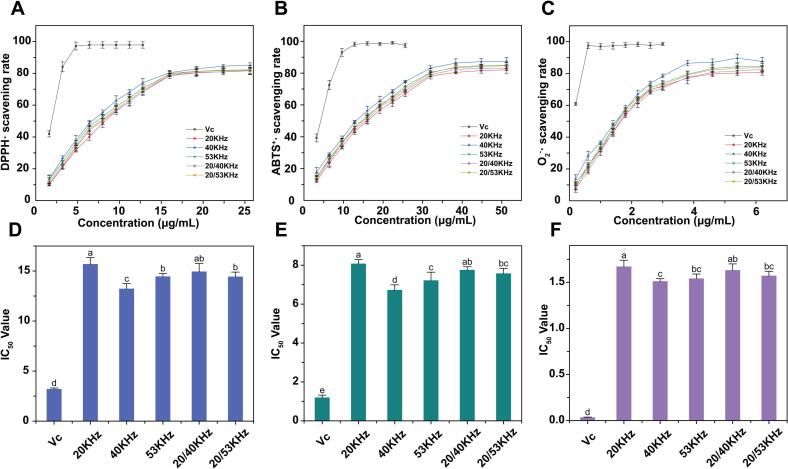
Antioxidant activity of RLMP obtained at different ultrasonic frequencies. Scavenging rates of (A) DPPH·, (B) ABTS^+^·,and (C) O_2_^–^· versus RLMP concentration; (D–F) corresponding IC_50_ values. Ascorbic acid (Vc) was used as the positive control.

Within the concentration range of 2–25 µg/mL, all RLMP samples prepared under different ultrasonic frequencies exhibited a dose-dependent increase in DPPH radical (DPPH·) scavenging activity (Fig. 5A), which plateaued at higher concentrations (80–85 %). The sample obtained using SFU at 40 kHz consistently demonstrated the highest scavenging efficiency, exceeding 60 % at just 12 µg/mL, with an half maximal inhibitory concentration (IC_50_) of approximately 13.22 µg/mL, lower than that of the 20 kHz group (15.68 µg/mL). Notably, the 40 kHz sample also exhibited the highest weight-average Mw (1.72 × 10^4^ Da), whereas samples obtained at 53 kHz and under DFU had lower Mw values (1.40–1.53 × 10^4^ Da). The higher Mw likely contributes to improved DPPH radical scavenging by providing more hydrogen-donating sites while maintaining favorable solubility [49].

#### 3.4.2 ABTS radical scavenging activity

Within the concentration range of 5–50 µg/mL, the ABTS radical (ABTS^+^·) scavenging rates of all samples increased with concentration, reaching a plateau of 80 %–88 % at 45–50 µg/mL (Fig. 5B). The 40 kHz sample exhibited the highest scavenging activity, achieving 87.3 % at 50 µg/mL, with an IC_50_ of 6.72 µg/mL, significantly outperforming the other groups. This superior performance may be attributed to the higher Mw of this sample, which enhances its electron-donating and hydrogen-donating capacities [39]. Conversely, the 53 kHz and 20/40 kHz dual-frequency samples, which exhibited a reduced Mw of 1.40 × 10^4^ Da, demonstrated lower ABTS radical scavenging efficiency.

#### 3.4.3 Superoxide anion scavenging activity

As shown in Fig. 5C, across the concentration range of 0.5–6 µg/mL, the superoxide anion (O_2_^–^·) scavenging activity of all samples increased rapidly and plateaued at 80–86 % near 6 µg/mL. The 40 kHz sample exhibited the lowest IC_50_ value of 6.72μg mL^–1^, 19–28 % lower than those of the 20 kHz, 53 kHz, and dual-frequency groups (*p* < 0.05 compared with all other groups, as determined by one-way ANOVA followed by Tukey's post-hoc test). This enhanced activity corresponds with its higher Mw, suggesting that a longer and more uniform polysaccharide backbone may facilitate O_2_^–^· binding and promote proton/electron donation [50]. Collectively, all three radical scavenging assays indicate that RLMP extracted using 40 kHz ultrasound possesses superior antioxidant activity. This enhanced activity is consistent with structural features observed in our characterization, including a higher Mw (1.72 × 10^4^ Da) and a more intact surface morphology as revealed by SEM analysis.

### 3.5 Intracellular antioxidant capacity

#### 3.5.1 Cytotoxicity assessment

**Fig. 6:**
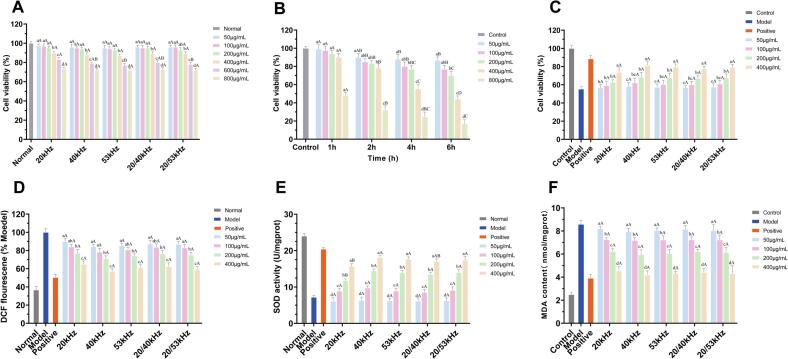
Intracellular antioxidant effects of RLMP in RAW 264.7 cells: (A) Cytotoxicity assay; (B) Establishment of an H_2_O_2_-induced oxidative stress model; (C) Protective effects of RLMP; (D) Intracellular ROS levels; (E) SOD activity; (F) MDA content. Data are presented as mean ± SD (n = 3). Different letters indicate significant differences (*p* < 0.05). Positive control: NAC (200 μg/mL); Normal: untreated control sample.

A compound is generally considered cytotoxic when cell viability falls below 80 % relative to untreated controls [51]. As shown in Fig. 6A, RLMP exhibited low cytotoxicity in RAW 264.7 cells at concentrations ranging from 50 to 400 μg/mL, with viability remaining above 80 %. However, at 800 μg/mL, cell viability decreased to 72.17 %, likely due to polysaccharide accumulation disrupting intracellular homeostasis. Consequently, concentrations of 50, 100, 200, and 400 μg/mL were selected for subsequent analyses.

#### 3.5.2 Protective effects against oxidative stress

Hydrogen peroxide (H_2_O_2_) was used to induce oxidative stress in RAW 264.7 cells [52]. As shown in Fig. 6B, low concentrations of H_2_O_2_ (50–100 μmol/L, 1 h) had minimal effects on cell viability, whereas higher concentrations and longer exposure significantly reduced viability. A concentration of 400 μmol/L H_2_O_2_ was selected based on preliminary dose–response experiments (50–800 μmol/L), which resulted in approximately 55.34 % reduction in cell viability, confirming the successful establishment of an oxidative stress model suitable for evaluating protective effects. RLMP treatment mitigated H_2_O_2_-induced cytotoxicity in a concentration-dependent manner (Fig. 6C). At 400 μg/mL, cell viability was restored to 81.56 % at 40 kHz, which was higher than that observed for 20 kHz (73.81 %), 53 kHz (79.43 %), 20/40 kHz (78.17 %), and 20/53 kHz (76.17 %). These results underscore the protective effect of RLMP against oxidative stress, particularly under 40 kHz treatment.

#### 3.5.3 Intracellular ROS levels

Intracellular ROS levels were measured to evaluate the antioxidant potential of RLMP at the cellular level. As illustrated in Fig. 6D, H2O2 treatment significantly increased ROS levels (*p* < 0.05), whereas RLMP administration suppressed ROS generation in a dose-dependent manner. At 400 μg/mL, RLMP reduced ROS levels by 35.19 % (20 kHz), 42.83 % (40 kHz), 39.02 % (53 kHz), 40.76 % (20/40 kHz), and 37.77 % (20/53 kHz) relative to the model group. The greatest ROS-reducing effect was observed with the 40 kHz sample, consistent with its superior performance in cell-free assays.

#### 3.5.4 Effects on SOD activity and MDA content

SOD plays a key role in neutralizing O_2_^–^· and maintaining redox homeostasis. By catalyzing the dismutation of superoxide into H_2_O_2_ and oxygen, SODs serve as a first line of antioxidant defense, preventing radical-induced macromolecular damage and preserving cellular redox equilibrium [53]. As shown in Fig. 6E, H2O2 exposure significantly suppressed SOD activity (7.20 U/mg protein) compared with the control (24.07 U/mg protein). RLMP treatment restored SOD activity in a dose-dependent manner, with the 40 kHz-treated sample exhibiting the highest activity (18.12 U/mg protein), followed by 53 kHz (17.69), 20/40 kHz (16.98), 20/53 kHz (17.41), and 20 kHz (15.67). The levels of MDA, a key biomarker of lipid peroxidation, were significantly elevated under oxidative stress (8.56 nmol/mL vs. 2.47 nmol/mL in controls). RLMP treatment markedly reduced MDA levels at 400 μg/mL: 47.1 % (20 kHz), 51.2 % (40 kHz), 49.5 % (53 kHz), 48.7 % (20/40 kHz), and 49.7 % (20/53 kHz) relative to the model group (Fig. 6F). These results indicate that RLMP alleviates lipid peroxidation and reinforces cellular antioxidant defenses, with the 40 kHz extract demonstrating the highest efficacy. Collectively, these findings suggest that RLMP confers robust protection against H_2_O_2_-induced oxidative stress by reducing ROS accumulation, promoting endogenous antioxidant enzyme activity, and limiting oxidative damage to cellular components. The enhanced bioactivity of the 40 kHz extract may stem from its optimal molecular structure and composition, particularly its higher molecular weight (1.72 × 10^4^ Da), as polysaccharide bioactivity is closely related to molecular structure parameters [54,55].

### 3.6 Single-factor test results for UAE

**Fig. 7:**
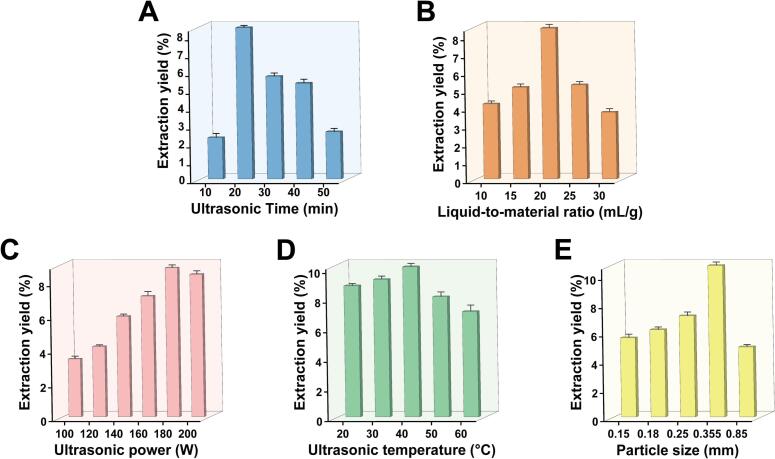
Effects of single factors on the extraction yield of RLMP: (A) ultrasound time, (B) liquid–solid ratio, (C) ultrasonic power, (D) ultrasonic temperature, and (E) particle size.

As shown in Fig. 7A, the extraction yield increased steadily with ultrasound time, reaching a maximum of 8.47 % after 30 min. Further prolongation of sonication led to a decline in yield. Ultrasound facilitates polysaccharide release by enhancing cavitation and mass transfer [20]; however, excessive exposure generates intense shear forces and localized heating [11], which can lead to polysaccharide degradation and a consequent reduction in yield. Thus, 30 min was determined to be the appropriate ultrasound time under the tested conditions.

Fig. 7B illustrates that increasing the liquid–solid ratio from 10:1 to 20:1 mL/g significantly improved the yield, reaching a maximum of 8.44 %. This enhancement is attributed to the greater availability of solvent, which facilitates the solubilization and diffusion of polysaccharides. However, further increasing the ratio to 30:1 diluted the ultrasonic energy density, thereby weakening cavitation effects and reducing extraction efficiency [56], while also increasing processing costs. Therefore, a 20:1 ratio was selected as the most suitable liquid–solid ratio among those tested.

The effect of ultrasonic power is depicted in Fig. 7C. Extraction yield increased with rising power, reaching a maximum at 180 W before declining. Moderate power enhances cavitation and mechanical effects, facilitating cell disruption and the release of compounds [57]. However, excessive power can lead to radical formation and over-degradation of polysaccharides. Therefore, 180 W was selected as the preferred ultrasonic power setting.

As shown in Fig. 7D, the extraction efficiency increased markedly between 20 °C and 40°C. Higher temperatures reduce solvent viscosity and surface tension, thereby enhancing cavitation. However, at 50°C, the yield declined, likely owing to the thermal degradation of polysaccharides [29]. Thus, 40°C was selected as the chosen ultrasonic temperature among those evaluated.

Fig. 7E shows that yield increased as particle size decreased from 0.85 mm to 0.355 mm, likely attributed to the enlarged surface area and shortened diffusion paths. Further reduction in particle size, however, caused a slight decrease in yield, possibly resulting from particle aggregation or hindered solvent penetration. Therefore, a particle size of 0.355 mm provided the most effective extraction performance.

### 3.7 Response surface experimental results

The BBD and the corresponding experimental results are presented in Table 3. A multivariate regression analysis was performed using a second-order polynomial (Eq. [11]) to model the relationship between the extraction yield (*Y*) and the independent variables:

(11) $$\begin{array}{c} Y=-167.345+1.657\times X_{1}+2.076\times X_{2}+1.044\times X_{3}+1.399\times X_{4}\\ +35.173\times X_{5}-0.0153\times X_{1}X_{2}-0.00161\times X_{1}X_{3}+0.000325\times X_{1}X_{4}+\\ 0.244\times X_{1}X_{5}+0.0018\times X_{2}X_{3}+0.00535\times X_{2}X_{4}-0.295\times X_{2}X_{5}-0.000725\\ \times X_{3}X_{4}-0.0904\times X_{3}X_{5}+0.0433\times X_{4}X_{5}-0.0190\times {X_{1}}^{2}-0.0492\\ \times {X_{2}}^{2}-0.00268\times {X_{3}}^{2}-0.0173\times {X_{4}}^{2}-19.488\times {X_{5}}^{2} \end{array}$$

The corresponding ANOVA is presented in Table 4. The model was highly significant (*F* = 55.6, *p* < 0.0001) and exhibited a non-significant lack-of-fit (*p* = 0.2328), indicating strong predictive accuracy. The coefficient of determination (*R*^2^ = 0.978) confirmed that 97.8 % of the variability was explained by the model. Among the variables, ultrasound time (*X_1_*) had the most pronounced effect (*p* < 0.001), followed by ultrasonic temperature, power, and particle size (*p* < 0.01). The liquid–solid ratio (*X_2_*) was not significant (*p* > 0.05). Significant interactions were observed for *X_1_X_2_* and *X_1_X_5_* (*p* < 0.001), as well as for *X_3_X_5_, X_2_X_5_, X_1_X_3_*, and *X_2_X_4_* (*p* < 0.01).

**Table 3:** Box-Behnken experimental design and its impact on the extraction yield of RLMP.

Run	*X_1_*	*X_2_*	*X_3_*	*X_4_*	*X_5_*	*Extraction yield*
1	20	20	180	40	0.25	7.15
2	30	20	180	50	0.85	8.11
3	20	25	180	40	0.55	7.98
4	30	15	180	50	0.55	7.25
5	30	25	160	40	0.55	8.37
6	30	20	200	30	0.55	7.81
7	30	20	180	50	0.25	7.36
8	30	15	180	30	0.55	7.66
9	30	20	180	30	0.85	7.03
10	30	25	180	50	0.55	8.12
11	30	15	180	40	0.85	8.14
12	30	25	180	40	0.25	8.31
13	40	20	160	40	0.55	9.11
14	30	20	180	40	0.55	10.35
15	40	20	180	30	0.55	7.52
16	30	25	180	30	0.55	7.46
17	20	20	180	50	0.55	6.47
18	30	20	180	30	0.25	6.8
19	30	20	180	40	0.55	10.88
20	30	15	200	40	0.55	7.91
21	30	25	180	40	0.85	7.79
22	20	20	200	40	0.55	7.04
23	40	15	180	40	0.55	9.06
24	40	20	180	40	0.85	8.16
25	30	20	180	40	0.55	10.86
26	30	20	180	40	0.55	10.71
27	30	20	160	30	0.55	7.76
28	40	20	180	50	0.55	8.09
29	40	25	180	40	0.55	7.87
30	30	25	200	40	0.55	8.06
31	30	20	200	40	0.85	7.39
32	30	20	160	40	0.85	8.83
33	40	20	180	40	0.25	6.52
34	20	15	180	40	0.55	6.12
35	30	20	200	40	0.25	8.02
36	30	20	180	40	0.55	10.76
37	40	20	200	40	0.55	7.89
38	30	20	160	50	0.55	8.49
39	30	15	180	40	0.25	6.89
40	20	20	180	40	0.85	5.86
41	20	20	180	30	0.55	6.03
42	30	20	200	50	0.55	7.96
43	20	20	160	40	0.55	6.97
44	30	15	160	40	0.55	8.94
45	30	20	180	40	0.55	10.74
46	30	20	160	40	0.25	7.29

**Table 4:** ANVOA analysis for RSM model.

Source	Sum of Squares	df	Mean Square	*F*-value	*p*-value	
Model	72.74	20	3.64	55.6	< 0.0001	significant
*X_1_*-Ultrasound time	7.02	1	7.02	107.36	< 0.0001	***
*X_2_*-Liquid-solid ratio	0.2475	1	0.2475	3.78	0.0631	
*X_3_*-Ultrasonic power	0.8464	1	0.8464	12.94	0.0014	**
*X_4_*-Ultrasonic temperature	0.893	1	0.893	13.65	0.0011	**
*X_5_*-Particle size	0.5513	1	0.5513	8.43	0.0076	**
*X_1_X_2_*	2.33	1	2.33	35.55	< 0.0001	***
*X_1_X_3_*	0.416	1	0.416	6.36	0.0184	*
*X_1_X_4_*	0.0042	1	0.0042	0.0646	0.8015	
*X_1_X_5_*	2.15	1	2.15	32.81	< 0.0001	***
*X_2_X_3_*	0.1296	1	0.1296	1.98	0.1716	
*X_2_X_4_*	0.2862	1	0.2862	4.38	0.0468	*
*X_2_X_5_*	0.7832	1	0.7832	11.97	0.002	**
*X_3_X_4_*	0.0841	1	0.0841	1.29	0.2676	
*X_3_X_5_*	1.18	1	1.18	18	0.0003	**
*X_4_X_5_*	0.0676	1	0.0676	1.03	0.3191	
*X_1_*2	31.66	1	31.66	484.07	< 0.0001	***
*X_2_*2	13.22	1	13.22	202.05	< 0.0001	***
*X_3_*2	10.02	1	10.02	153.17	< 0.0001	***
*X_4_*2	26.16	1	26.16	399.98	< 0.0001	***
*X_5_*2	26.85	1	26.85	410.44	< 0.0001	***
Residual	1.64	25	0.0654			
Lack of Fit	1.45	20	0.0726	1.97	0.2328	not significant
Pure Error	0.1841	5	0.0368			
Cor Total	74.38	45				
R^2^	0.978					
Adjusted R^2^	0.9604					
Predicted R^2^	0.9184					
Adequacy Precision	28.1706					

Note: * indicates *p*-value < 0.05, ** indicates *p*-value < 0.01, *** indicates *p*-value < 0.001.

**Fig. 8:**
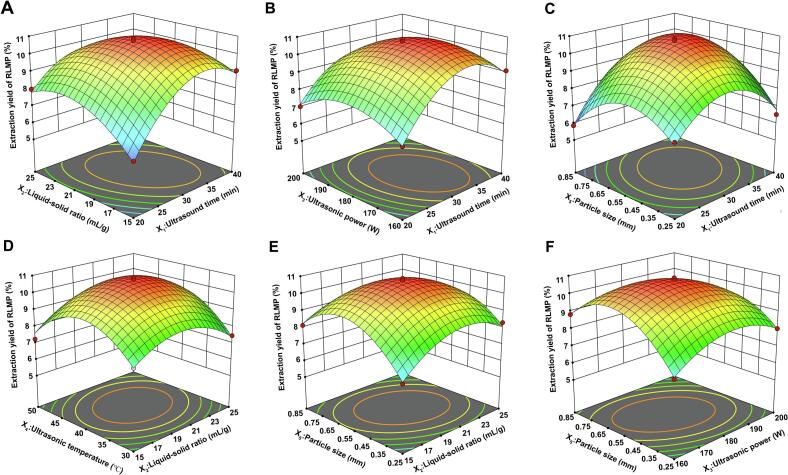
3D response surface plots illustrating the interactions between pairs of variables: (A) ultrasound time and liquid–solid ratio; (B) ultrasound time and ultrasonic power; (C) ultrasound time and particle size; (D) liquid–solid ratio and ultrasonic temperature; (E) liquid–solid ratio and particle size; (F) ultrasonic power and particle size.

To visualize the simultaneous influence of any two variables on the extraction yield of RLMP, the remaining factors were held at their central (zero) levels, and three-dimensional response-surface plots were generated from the fitted regression model (Fig. 8A–F). In these plots, a steeper surface indicates greater sensitivity of the response to the corresponding factor pair, whereas the concentric contour center represents the predicted optimum conditions for RLMP recovery. Inspection of Fig. 8 shows that ultrasound time is the primary determinant of extraction efficiency, followed, in descending order, by ultrasonic temperature, ultrasonic power, and particle size; the liquid–solid ratio has only a minor effect. Among the interaction terms, the surfaces for ultrasound time versus liquid–solid ratio and ultrasound time versus particle size are the steepest, indicating that these combinations exert the most pronounced synergistic effects on RLMP yield.

Based on Eq. (11), Design Expert software optimization identified the optimal conditions for RLMP extraction through numerical analysis of the response surface and the circle′s center shown in the contours, the optimal conditions for RLMP extraction were determined as follows: ultrasound time of 32.275 min, liquid–solid ratio of 19.751 mL/g, ultrasonic power of 176.287 W, ultrasonic temperature of 40.792°C, and particle size of 0.592 mm. Under these conditions, the predicted maximum RLMP extraction yield was 10.83 %. For experimental practicality, the parameters were rounded to 32 min, 20 mL/g, 180 W, 41°C, and 0.6 mm (corresponding to standard 30-mesh sieve size commonly used in powder processing). Using these adjusted conditions, the actual RLMP extraction yield reached 10.65 %, which falls within the 95 % confidence interval of the predicted value. However, given the limitations of RSM in handling complex nonlinear relationships and the potential for local optima, PSO-SVR was subsequently employed to explore the global optimum.

### 3.8 PSO-SVR optimization of polysaccharide extraction process

**Fig. 9:**
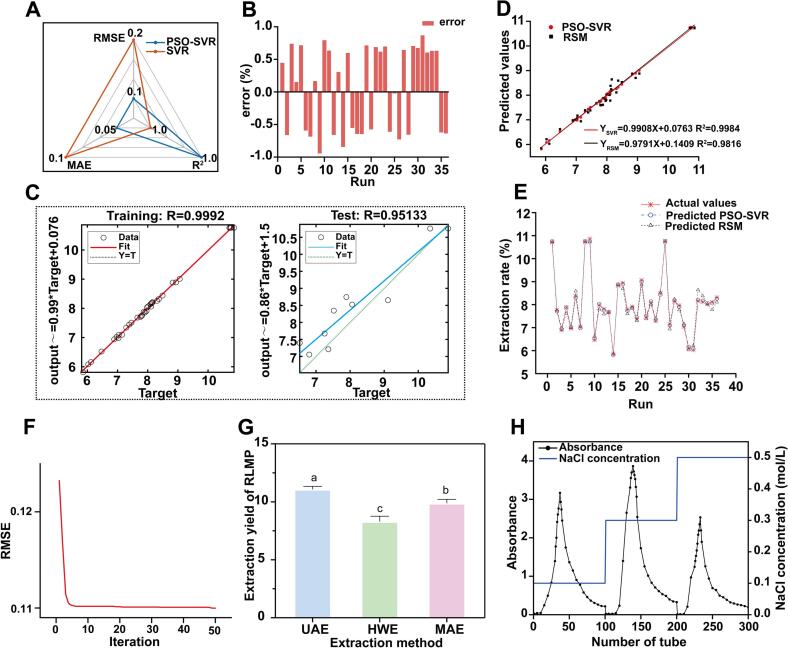
Performance evaluation and prediction using PSO-SVR: (A) radar chart, (B) prediction errors, (C) parity plots, (D) model comparison, (E) run-wise predictions, (F) RMSE convergence, and (H) elution profile from DEAE-cellulose chromatography.

While RSM provided valuable insights into the extraction process, its inherent assumption of quadratic relationships may not fully capture the complex nonlinear interactions involved in ultrasonic extraction. Therefore, SVR modeling combined with PSO optimization was employed to identify potentially superior extraction conditions. Based on BBD results, SVR modeling was used to predict RLMP extraction yield. As shown in Fig. 9A, PSO optimization statistically significantly improved SVR model performance (*F =* 1.97, *p* < 0.05): *R*^2^ increased from 0.9707 to 0.9983, RMSE decreased by 76.2 % from 0.2108 to 0.0501, and MAE declined by 49.8 % from 0.0953 to 0.0478, representing a 94 % reduction in unexplained variance and substantial improvement in prediction accuracy for practical applications. Prediction errors remained within ± 1 % (Fig. 9B), and parity plots confirmed an excellent fit for both training and test datasets (Fig. 9C). These improvements in performance metrics reflect an optimized bias–variance trade-off achieved through parameter tuning, providing a robust solution for complex nonlinear regression problems. As illustrated in Fig. 8D, the PSO-SVR model yielded the regression equation *Y_SVR_* = 0.9908*X* + 0.0763 and R^2^ = 0.9984, indicating an almost ideal 1:1 correspondence between predicted and experimental extraction yields. By contrast, the RSM model produced *Y_RSM_* = 0.9791*X* + 0.1409 and *R*^2^ = 0.9816, with systematic underprediction at low yields and overprediction at higher yields. This advantage of the PSO-SVR model was further supported by the run-wise comparison (Fig. 8E): PSO-SVR predictions consistently aligned with the actual extraction rates across 37 independent validation runs, whereas RSM estimates showed more pronounced deviations, particularly at the extremes. Fig. 8F presents the optimization results obtained using the PSO-SVR model. The algorithm converged after 49 iterations, yielding the following optimal conditions, as indicated by PSO: ultrasound time of 34.2214 min, liquid–solid ratio of 18.8955 mL/g, ultrasonic power of 178.2917 W, ultrasonic temperature of 41.3297°C, and particle size of 0.3741 mm. Under these conditions, the predicted maximum RLMP yield was 10.98 %. For experimental practicality, the parameters were rounded to 34 min, 19 mL/g, 180 W, 41°C, and 0.355 mm (corresponding to standard 45-mesh sieve size commonly used in powder processing). Using these adjusted conditions, the actual RLMP extraction yield reached 11.07 %, falling within the 95 % confidence interval of the predicted value.

These results demonstrate the robustness, predictive accuracy, and generalization capability of the PSO-SVR model for optimizing polysaccharide extraction under nonlinear conditions.

### 3.9 Comparison of extraction methods

As shown in Fig. 9G, the extraction yield of RLMP obtained using UAE (10.95 % ± 0.09 %) was significantly higher than that achieved by MAE (9.87 % ± 0.28 %) and HWE (8.30 % ± 0.37 %). Statistical analysis confirmed that the yield from UAE was significantly higher than that from MAE (*p* < 0.05) and HWE (*p* < 0.01). These results indicate that UAE markedly improves the efficiency of RLMP extraction under the tested conditions.

### 3.10 Isolation and purification of RLMP

RLMP was further purified using DEAE-52 cellulose anion-exchange chromatography to isolate the target fraction. As shown in Fig. 9H, stepwise elution with deionized water and NaCl solutions at 0.1, 0.3, and 0.5 M produced a single major peak corresponding to a neutral polysaccharide fraction eluted with 0.3 M NaCl. This major fraction was collected, extensively dialyzed against distilled water overnight, and lyophilized to yield a white, flocculent polysaccharide powder, designated RLMP-2, which contained 93.34 % total carbohydrates as determined by the phenol–sulfuric acid method.

### 3.11 NMR

**Fig. 10:**
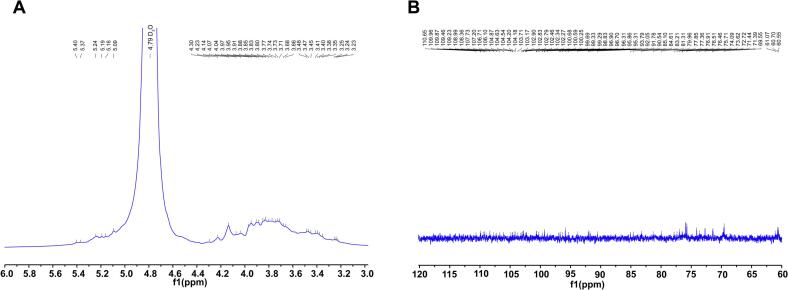
NMR spectra of RLMP-2: (A) ^1^H NMR, (B) ^13^C NMR.

The ^1^H NMR spectrum of RLMP-2 (Fig. 10A) displayed five anomeric proton signals in the 5.40–5.09 ppm range, along with a broad signal envelope between 4.30 and 3.23 ppm. Signals above 5.00 ppm are characteristic of α-anomers, whereas the 5.09 ppm signal indicates a β-configuration, confirming the coexistence of both α- and β-glycosidic linkages [58]. Downfield shoulders near 4.25 ppm suggest O-substitution, indicative of branching. The ^13^C NMR spectrum (Fig. 10B) showed resonances in the 60–110 ppm range, consistent with a neutral polysaccharide lacking UAs. Anomeric carbon peaks (105.9–98.3 ppm) confirmed the presence of β-D- and α-D-pyranosyl residues, while signals between 86 and 70 ppm corresponded to substituted and unsubstituted ring carbons. Sharp peaks at 60–62 ppm were attributed to the C-6 carbons of hexose units, with no evidence of C-6 substitution [59]. Collectively, these spectral features confirm that RLMP-2 is a heteropolysaccharide comprising multiple monosaccharide units, containing both α- and β-glycosidic linkages, and exhibiting potential branching.

## 4 Discussion

Extraction is a crucial step in isolating bioactive compounds from plant materials, with efficiency and product quality strongly dependent on technical parameters. Under optimized UAE conditions, an 11.07 % yield of RLMP was achieved, which was significantly higher than that obtained through MAE and HWE. However, this yield was slightly lower than that reported for *R. laevigata* polysaccharide extracted via microwave or microwave-assisted aqueous two-phase systems [33,35]. This discrepancy can be partly attributed to methodological differences: in this study, extraction yield was calculated based on alcohol-precipitated polysaccharide, whereas other studies often report yields based on crude extracts, limiting direct comparability.

Ultrasonic frequency significantly influences the monosaccharide composition and Mw distribution of polysaccharides, likely owing to the differential sensitivity of glycosidic linkages to acoustic cavitation [16,18,19]. According to the Rayleigh–Plesset equation, ultrasonic frequency modulates bubble dynamics, with higher frequencies generating smaller bubbles that collapse with lower intensity [20]. At 40 kHz, the intense cavitation selectively cleaves the more accessible side chains of polysaccharides, leading to an increased proportion of linear glucose polymers [16,18,37]. Given the predominance of glucose in RLMP, glucose content was employed as a key indicator to assess frequency-dependent changes in monosaccharide composition. The retention of Mw at intermediate frequencies suggests an optimal window in which mass transfer is enhanced without excessive formation of hydroxyl radicals that could trigger random chain scission [10,15]. In contrast, higher frequencies produce a greater number of milder cavitation events, which may be insufficient to penetrate crystalline cellulose regions effectively but can still induce oxidative degradation of more accessible chains [15,28,34]. Dual-frequency systems can generate heterogeneous acoustic fields with alternating pressure zones, leading to incomplete extraction [60]. These findings provide a theoretical framework for designing targeted extraction strategies that consider the hierarchical architecture of plant cell walls and the mechanical properties of specific polysaccharide fractions. The most effective frequency identified in this study (40 kHz) aligns with findings from other plant polysaccharide extraction studies [27,32]. However, our finding that dual-frequency treatments underperformed contrasts with some reports showing synergistic effects in other systems [[15], [16], [17],^61^], highlighting the importance of species-specific and matrix-dependent optimization.

RSM is a widely used statistical tool for process optimization. However, its inherent quadratic assumptions often constrain its ability to model the complex nonlinear interactions present in UAE systems. In comparison, the hybrid PSO-SVR approach demonstrated superior performance in optimizing RLMP extraction. This enhancement can be attributed to the SVR's kernel function, particularly the radial basis function, which effectively captures the nonlinear relationships among ultrasonic frequency, power, and time [62]. SVR adapts directly to the underlying process dynamics from the data. Moreover, the global search capability of PSO mitigates the risk of local optima that commonly hinder gradient-based methods [63], thereby further enhancing the model's optimization efficiency. This capability is particularly valuable in ultrasonic extraction, where cavitation, heat, and mass transfer interact in complex, nonlinear ways that traditional models often fail to capture.

Despite providing valuable insights into the frequency-dependent behavior of RLMP extraction, several limitations remain. The examined frequency range (20–53 kHz) represents a relatively narrow segment that may not capture optimal conditions for different polysaccharide fractions or extraction matrices. Investigating higher frequencies (> 100 kHz), triple-frequency combinations, or frequency-sweep protocols could reveal additional cavitation mechanisms and improve extraction selectivity. A critical limitation is the absence of direct acoustic field characterization. Future studies should incorporate acoustic field mapping and cavitation quantification techniques, such as sonochemiluminescence or hydrophone measurements, to establish more direct correlations between acoustic parameters and extraction outcomes. Additionally, scaling from laboratory to industrial applications presents significant challenges, particularly maintaining uniform acoustic field in large reactors due to sound attenuation and standing wave formation. Potential solutions include phased-array transducer systems, continuous-flow reactors, and hybrid technologies integrating ultrasound with other green extraction methods.

From a bioactivity perspective, this study focused specifically on antioxidant properties, which represents only one aspect of polysaccharide therapeutic potential. Future investigations should systematically evaluate anti-inflammatory and immunomodulatory effects, as well as potential prebiotic activities, to provide a comprehensive bioactivity profile. Additionally, structure–activity relationship studies correlating specific molecular features (molecular weight, glycosidic linkage patterns) with biological activities would enhance mechanistic understanding and support rational extraction optimization.

## 5 Conclusion

This study systematically investigated ultrasonic frequency effects on Rosa laevigata polysaccharide extraction, revealing that 40 kHz single-frequency treatment achieved improved extraction efficiency and product quality. The frequency-dependent extraction mechanism was preliminarily investigated, showing preferential effects on structural characteristics and antioxidant activity. PSO-SVR modeling proved superior to traditional RSM, enabling process optimization that achieved 11.07 % extraction yield. The extracted polysaccharides demonstrated notable antioxidant activity and cellular protective effects. Overall, this work provides novel insights into frequency-dependent ultrasound extraction mechanisms and highlights the antioxidant potential of RLMP, establishing a foundation for future scale-up studies and industrial process development for nutraceutical applications.

## CRediT authorship contribution statement

**Yuyuan Duan:** Writing – original draft, Investigation, Formal analysis. **Shuting Wang:** Visualization, Investigation. **Xiaorong Zhang:** Investigation, Data curation. **Huimei Zhang:** Investigation. **Huizhu Wang:** Validation, Supervision, Resources, Funding acquisition, Conceptualization. **Shuai Chen:** Supervision, Project administration, Methodology, Funding acquisition, Conceptualization.

## Funding

This work was supported by the 10.13039/501100010211Department of Education of Jilin Province (JJKH20200256KJ) and the Jilin Provincial Science and Technology Department (JDZJ202501ZYTS159), China.

## Declaration of competing interest

The authors declare that they have no known competing financial interests or personal relationships that could have appeared to influence the work reported in this paper.
