# Supplementary material for: Mechanistic insight into the frequency-dependent ultrasound-assisted extraction of Rosa laevigata Polysaccharides: Structure, antioxidant activity, and process optimization
Source: Ultrason Sonochem. 2025 Sep 19;121:107577. doi: 10.1016/j.ultsonch.2025.107577 (PMC12492013; doi:10.1016/j.ultsonch.2025.107577)
Supplement: Supplementary Data 1 [file mmc1.docx]

**Table S1.** **Thermal transition temperatures and enthalpy changes of RLMP extracted under different ultrasonic frequency conditions**

|  | ***T***_1_(℃) | ***T***_2_(℃) | ***T***_3_(℃) | ***T***_4_(℃) | ***T***_5_(℃) |  | Δ***H***_12_(kJ/g) | Δ***H***_23_(kJ/g) | Δ***H***_34_(kJ/g) | Δ***H***_45_(kJ/g) |
| --- | --- | --- | --- | --- | --- | --- | --- | --- | --- | --- |
| 20kHz | 56.91 | 130.85 | 190.20 | 293.08 | 554.19 |  | 2.48 | -11.05 | -8.16 | -33.35 |
| 40kHz | 58.04 | 129.96 | 185.33 | 290.93 | 554.19 |  | 2.26 | -8.48 | -8.12 | -35.60 |
| 53kHz | 46.94 | 128.16 | 187.61 | 290.93 | 554.19 |  | 1.82 | -13.94 | -16.63 | -77.49 |
| 20/40kHz | 36.64 | 125.55 | 187.38 | 292.03 | 564.32 |  | 7.63 | -14.31 | -12.68 | -56.13 |
| 20/53kHz | 51.04 | 126.71 | 187.59 | 292.24 | 566.59 |  | 2.27 | -12.34 | -11.98 | -52.62 |
